# 

**Published:** 1882-09

**Authors:** 


ESTABLISHED IN 1855.

OLD SERIES.
Vol. XX—No. 6.

SEPTEMBER, 1882.

NEW SERIES.

Vol. I—No. 12.

S.G.O. INDEXED

### ATLANTA
### MEDICAL REGISTER.

(*ATLANTA MEDICAL AND SURGICAL JOURNAL.*)

EDITED BY

JAMES B. BAIRD, M.D.

H. H. DICKSON, PUBLISHER. TERMS, $2.50 A YEAR IN ADVANCE.

ATLANTA, GEORGIA:

*H. H. DICKSON, BOOK AND JOB PRINTER AND BOOKBINDER.*

1882.

